# Identification and Differentiation of Non-Hemolytic *Listeria monocytogenes* from Food Processing Environments Using MALDI-TOF MS

**DOI:** 10.3390/molecules30143049

**Published:** 2025-07-21

**Authors:** Barbara Szymczak

**Affiliations:** Department of Applied Microbiology and Human Nutrition Physiology, Faculty of Food Sciences and Fisheries, West Pomeranian University of Technology, Papieża Pawła VI 3, 71-459 Szczecin, Poland; barbara.szymczak@zut.edu.pl; Tel.: +48-449-65-43

**Keywords:** *L. monocytogenes*, hemolysin-negative isolates, MALDI-TOF MS, food safety, biomarker, peptides

## Abstract

Out of 2495 samples, *L. monocytogenes* was isolated from 262 (10.5%). Among these, 30 isolates (11.5% of the 262) exhibited unique phenotypic and genetic characteristics compared to reference strains. Hemolysin-negative *L. monocytogenes* isolates have been increasingly reported in recent years and are challenging to identify due to their altered phenotypic traits and limitations of standard microbiological methods. This study aimed to evaluate the performance of MALDI-TOF MS in identifying and differentiating 30 hemolysin-negative and hemolysin-positive *L. monocytogenes* isolates and 12 reference strains, using both a commercial Bruker database and a proprietary in-house database developed from newly characterized isolates. The Bruker database correctly identified only 21% of the environmental isolates, misclassifying most as *L. innocua*, and showed 83.3% accuracy for reference strains. In contrast, the in-house database achieved 96.6% and 100% accuracy for the environmental and reference strains, respectively. Statistical methods, including hierarchical clustering, heatmaps, PCA, and Pearson correlation, revealed grouping based on phenotypic traits and origin, with key peptides influencing classification. Biomarkers linked to hemolysis and antibiotic resistance differentiated the environmental isolates from reference strains. These findings highlight the need for the development of customized spectral databases to improve the detection of *L. monocytogenes* in food safety monitoring.

## 1. Introduction

Listeriosis is one of the most serious and severe foodborne diseases caused by the bacteria *Listeria monocytogenes (L. monocytogenes)*. Although rare, with 0.1 to 10 cases per 1 million people per year, its high mortality rate, approximating 30%, makes it a serious public health concern [1]. Of the 29 species of *Listeria*, only *L. monocytogenes* is pathogenic to humans; however, isolated cases of listeriosis have been reported following infection with *L. innocua* and *L. ivanovii* [2,3,4].

The main cause of listeriosis is the consumption of *L. monocytogenes*-infected foods, such as meat, eggs, milk, seafood, fruits, and manure-fertilized vegetables [5,6]. *L. monocytogenes* has 14 serotypes divided into four phylogenetic groups, but more than 98% of listeriosis cases are ascribed to serotypes 1/2a, 1/2b, and 4b [7]. Due to the genetic heterogeneity of *L. monocytogenes*, serotyping is crucial for epidemiological surveillance, but is not required for identification [8]. Reference methods include time-consuming tests for the genus *Listeria* sp. (Gram staining, motility, catalase, and oxidase test) and the species *L. monocytogenes* (ability to degrade sugars and hemolysis, CAMP test). In contrast, advanced techniques, such as *Listeria* API, PCR, and 16S rRNA sequencing, are costly and complex, which limits their routine use by food manufacturers, particularly in the case of foods with short shelf-life. Recent studies have reported atypical *L. monocytogenes* isolates, including serotype 4h, which do not ferment rhamnose [9,10]; however, their identification is not addressed in the ISO 11290-1:2017 standard method [8].

The increasing incidence of listeriosis in Europe has led to a zero-tolerance policy for *L. monocytogenes* in ready-to-eat food (RTE food) [11]. Contamination with *L. monocytogenes* causes significant financial losses due to product recalls and undermines consumer trust, making rapid and reliable identification of *L. monocytogenes* essential. An alternative to conventional methods is matrix-assisted laser desorption ionization–time-of-flight mass spectrometry (MALDI-TOF MS) [12]. This technique is considered reliable, fast, and cost-effective for the routine identification of pathogens in clinical, food, and environmental samples. MALDI-TOF analyzes bacterial protein profiles (2–20 kDa), mainly ribosomal and core metabolic proteins, which form a specific bacterial fingerprint compared to protein profiles in reference spectral libraries [13,14,15]. MALDI-TOF MS effectively differentiates *Listeria* sp., even among pathogenic strains, and allows for database expansion, which improves identification effectiveness [16,17,18].

Despite its many advantages, the differentiation of *Listeria* sp. remains a challenge due to their genetic relatedness, gene transfer, and phenotypic similarity to other *Listeria* sp. [17]. Additionally, the increasing frequency of atypical *L. monocytogenes* isolates [5,9] highlights the need for further research harnessing MALDI-TOF MS, depending on the isolation environment and phenotypic characteristics of the new isolates. Non-hemolytic *L. monocytogenes* strains, which lack the β-hemolysis activity, being a key feature enabling differentiation of *L. monocytogenes* from other *Listeria* species, are increasingly frequently isolated from various environments, including food, animals, and clinical samples [19]. These isolates may harbor key virulence genes (e.g., *prfA*, *inlA*, *actA*, *plcA*, and *mpl*) despite suppressed or silenced expression, often influenced by environmental stress or regulatory mutations (e.g., temperature, pH, and nutrient availability). Therefore, the absence of hemolytic activity does not unequivocally indicate a loss of pathogenic potential of these isolates, and their greater resistance to stressors, disinfectants, and antibiotics may indicate their severe threat to the food industry [20,21]. Their atypical phenotype poses a diagnostic challenge since they may be misidentified as non-pathogenic species using standard phenotypic methods. This highlights the need for harnessing advanced identification techniques, such as MALDI-TOF MS.

The present study involved a detailed analysis of 30 unique *L. monocytogenes* isolates conducted by means of the MALDI-TOF MS method—for the first time on a global scale. The isolates originated from various environments, making this study a valuable contribution to research on the diversity and variability of this bacterium.

This study included a comparison of the isolate hemolytic activity, antibiotic susceptibility, and other phenotypic characteristics, as described in detail in the publication by Szymczak [5]. This analysis enables a deeper understanding of *L. monocytogenes* properties and may support the development of new identification methods—particularly for hemolysin-negative isolates, which, despite their incapability for hemolysis, may pose a significant threat in the food industry. The current ISO standard [8] does not provide any specific identification approach to such isolates. Therefore, the findings from this study may serve as a starting point for further research and contribute to the improvement of existing diagnostic methods. Therefore, the aim of this study was to compare the identification effectiveness of hemolysin-negative *L. monocytogenes* isolates using MALDI-TOF MS, employing both the Bruker DB and an in-house database, while considering the isolation environment and phenotypic traits.

## 2. Results and Discussion

### 2.1. Identification of L. monocytogenes Reference Strains and Environmental L. monocytogenes Isolates

The Bruker database enabled highly probable identification of 10 (83.3%) of the 12 *L. monocytogenes* reference strains from different serotypes at the species level (score 1.737–2.263). The remaining two *L. monocytogenes* reference strains, 1/2a and 4e, were incorrectly identified as *L. innocua* (score 1.708 and 1.841) (Table 1). Among the 30 environmental isolates, only 7 (21%) were confirmed as *L. monocytogenes* (score 1.762–2.183), whereas 20 (60%) were identified as *L. innocua* (score 1.737–2.245), 2 isolates as *L. ivanovii* (1.745) and *L. seeligeri* (1.741), and 1 isolate remained unidentified (Table 2). The isolation environment of *L. monocytogenes* did not affect the identification effectiveness, with average Biotyper scores of 1.893 for swabs, 1.933 for fruits, 1.940 for vegetables, 1.954 for soil, and 1.993 for RTE food (Table 2). The average Biotyper score for all *L. monocytogenes* isolates was 1.947. Previous phenotypic and genetic studies confirmed the identification of the same 30 isolates as *L. monocytogenes* [5]. Compared to the reference strains, the environmental isolates were non-motile, and 25 out of the 30 analyzed were non-hemolytic (Table 3). Differences in the effectiveness of identification using MALDI-TOF MS may be due to various sample preparation methods, the use of various media, and different incubation times [22,23]. It has been demonstrated that a 24 h incubation on BHI Agar ensures over 90% identification effectiveness at the genus level [23]. After 48 h of incubation on PALCAM (Polymyxin Acriflavine Lithium Chloride Ceftazidime Esculin Mannitol) and ALOA (Agar Listeria Ottaviani and Agosti) media, the identification effectiveness was 50% at the genus level and 37% at the species level. Different environments can affect the phenotypic traits of bacteria, which may interfere with their identification using MALDI-TOF MS. However, there is a paucity of studies indicating the impact of bacterial environment on the accuracy of identification [24,25]. An additional limitation is the optimization of the MALDI-TOF MS database primarily for clinical isolates rather than environmental ones [26]. Despite these drawbacks, Thouvenot et al. [27] achieved 100% identification effectiveness for 363 hemolysin-positive *L. monocytogenes* isolates from clinical and food samples.

In the next step, the 42 protein profiles obtained were added to the Biotyper database, creating an in-house database, and the identification of *L. monocytogenes* reference strains and environmental *L. monocytogenes* isolates was repeated. The expanded database enabled highly probable identification of 12 reference strains of *L. monocytogenes* at the species level (score 2.602–2.778) (Table 1). Then, 29 (96.6%) out of the 30 isolates from various food production environments were identified as *L. monocytogenes* (Table 2). The average score for all isolates was 2.218, which was comparable across the isolates from soil (score 2.221), fruits (score 2.123), vegetables (score 2.217), and RTE food (score 2.217), with swabs yielding a significantly higher score of 2.305. Despite using the in-house database, the same *L. monocytogenes* isolate (no. 111) was once again not identified, with a score of 1.686.

The results showed that the use of the in-house database significantly improved the identification effectiveness of reference strains from 83.3% to 100% and of the environmental isolates from 21% to 96.6%. Similarly, Karbiwnyk et al. [28] improved the reliability of *L. monocytogenes* identification from 64% to 90% by adding peptide profiles from non-clinical strains to the database. Dos Reis et al. [29] demonstrated that atypical *Listeria* sp. could be identified using MALDI-TOF MS upon extending the database with protein profiles from local sources. In the study by Kouderka et al. [13], the identification effectiveness was increased from 73.2% (Biotyper) to 93.8% (ClinPro Tool) after the MALDI-TOF MS database had been supplemented with 112 strains from food and the environment. In contrast, Rychert et al. [30] found that 7 out of 45 *L. monocytogenes* strains were not identified, and 4 of them were only confirmed to belong to the *Listeria* genus. When analyzing 16 bacterial genera and 73 Gram-positive species from the Firmicutes group (*Actinomyces*, *Bacillus*, *Corynebacterium*, *Lactobacillus*, *Listeria*, *Nocardia, Propionibacterium*, *Nocardia*, *Erispelothrix*, *Gardnerella*, *Paenibacillus*, and *Rhodococcus*), Farfour et al. [31] correctly identified 98.5% of the strains at the species level, while 1.2% were not identified even at the genus level. Therefore, the authors argued that the continuous curation of MALDI-TOF MS databases should be carried out as a dynamic process. In turn, Li et al. [32] proposed harnessing machine learning to improve the identification effectiveness of atypical bacterial strains.

**Figure 1:**
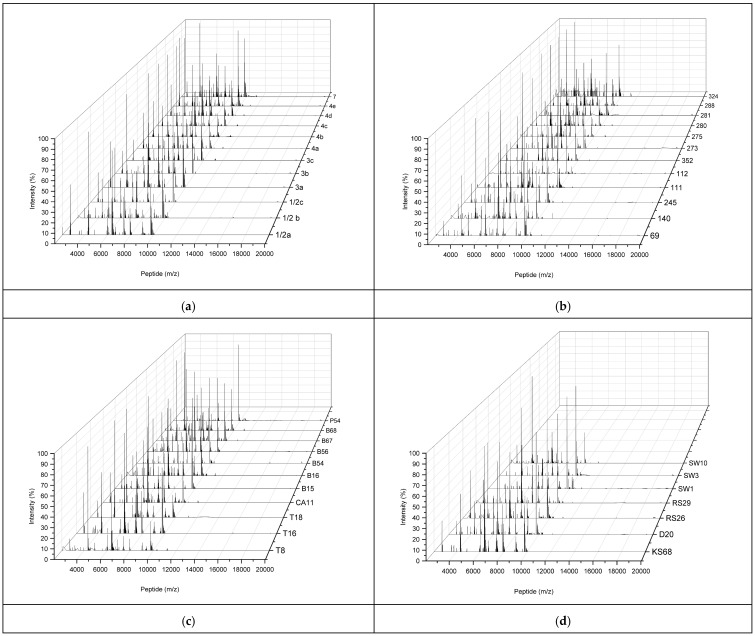
Protein profiles of *L. monocytogenes* reference strains (**a**) and *L. monocytogenes* isolates from soil (**b**), fruit and vegetables (**c**), and RTE food and swabs (**d**). The *X*-axis shows the mass-to-charge ratio (*m*/*z*), while the *Y*-axis shows the peptide intensity (%). The isolated numbers are described in Table 3.

Due to the incorrect identification of two (16.7%) reference strains and 20 (66.7%) environmental isolates as *L. innocua* using the Bruker database, identification was performed using specific peptides (biomarkers). Barbuddhe et al. [33] demonstrated that peptides with molecular masses of 7922 and 7941 Da enabled differentiation between *L. innocua* and *L. monocytogenes.* The 7922 Da peptide was present only in T16, KS68, and D20 isolates, two of which were identified as *L. innocua* and one as *L. monocytogenes* by MALDI-TOF MS (Table 2), while the 7941 Da peptide was found in 7 out of the 12 reference strains of *L. monocytogenes* and in 9 out of the 30 atypical *L. monocytogenes* isolates (Figure 1). Abdelhamed et al. [34] identified 10 peptides as potential biomarkers for species-level identification of *L. monocytogenes*, of which 4326, 4878, 7405, 9040, and 9754 Da were present in all reference strains of *L. monocytogenes*, while the remaining 5 peptides varied according to the serotype of the strain (Figure 1). Among the environmental isolates, all contained peptides 7405, 9754, and 4326 Da (except for isolates 112, P54) and 4878 Da (except for isolates 111, T18), while peptides 3194, 5302, 6718, 6863, 9040, and 11,182 Da were found in 12, 0, 8, 17, 24, and 0 isolates, respectively. To improve the identification effectiveness, the possibility of peak shifts in the range of 0.2–2.2 Da was considered, addressing the problem of stability and variation coefficient in the MALDI-TOF MS method [14,35,36] and taking into account the recommendation that biomarkers used in the identification of *Listeria* should differ by at least 1 Da for every 1000 Da of peptide mass [23,28]. Despite this, the use of a single peptide to differentiate *Listeria* species, as well as the use of the Bruker database, failed to ensure full identification effectiveness.

### 2.2. Analysis of Protein Profiles in Terms of the Isolation Environment

**Figure 2:**
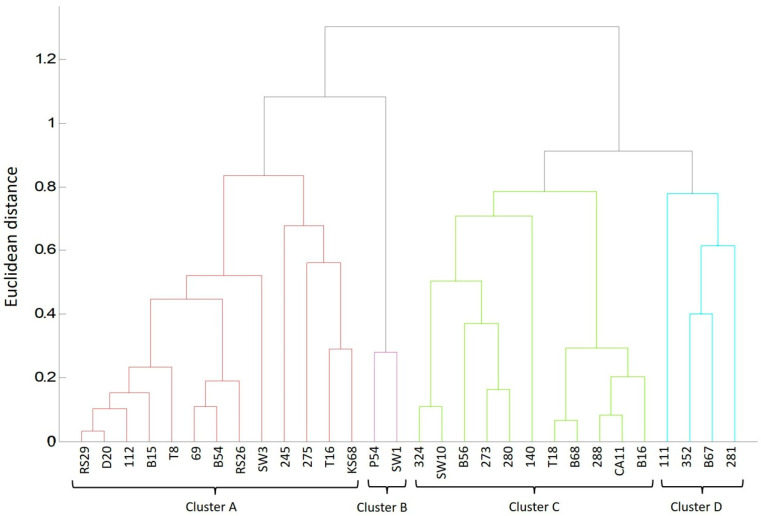
Mass spectra profile dendrogram generated by MALDI Biotyper 3.1 to determine correlations between 30 isolates of *L. monocytogenes* from different food production environments.

At a dissimilarity level of 1.3 (maximum dissimilarity), dendrogram analysis classified environmental *L. monocytogenes* isolates into four clusters (A-D) (Figure 2). The largest cluster, A, contained 13 isolates: 4 isolates from soil, 4 from fruits and vegetables, 3 from sprouts, 1 from dumplings, and 1 from a line swab. In cluster A, there were four correlations between the isolate and its environmental source. Hemolysin-negative isolates 112 and 245 were isolated from manure-fertilized soil where vegetables were grown, which in turn were sources of isolates B15 and B54 (Table 3). Isolate D20 (dumplings) is derived from the same processing line as isolate SW3 (swab), whereas isolates RS26 and RS29 (radish sprouts) originated from the same producer. In cluster A, as many as eight isolates were grouped at a low dissimilarity level of 0.2–0.3 Euclidean units, indicating their high similarity, although the isolates from the same environment clustered at distances greater than 0.5. Cluster B included two isolates (P54 and SW1) from different environments, which exhibited high dissimilarity, suggesting greater differences between them. Cluster C included 11 isolates from soil, fruits, and vegetables, and one swab (Figure 2). Isolate B16 was derived from beets grown in soil containing isolate 280, while isolate T18 was from strawberries grown in areas where hemolysin-negative isolate 324 was detected (Table 3). In cluster C, similar to cluster A, most isolates formed small subgroups at low dissimilarity levels, but the differences between the subgroups were greater than in cluster A. Cluster D grouped four isolates from soil and vegetables, with isolate B67 (beet) originating from the same soil as isolate 352, but they only merged on the dendrogram at a distance of 0.4.

The grouping analysis revealed all 12 correlations between the isolates from food, soil, and swabs. The lack of clustering of the remaining 18 *L. monocytogenes* isolates was also justified (Figure 2), as they originated from unrelated environments (Table 3). No similar studies available in the literature have covered such a large group of hemolysin-negative *L. monocytogenes* isolates from diverse environments. However, Pyz-Lukasik et al. [14] demonstrated that cluster analysis allowed for the grouping of *L. monocytogenes* isolates from cheeses derived from different production plants based on their protein profiles, regardless of sampling time. These findings confirm that cluster analyses are more effective in grouping *L. monocytogenes* isolates based on protein profiles than on their ability to ferment sugars, their antibiotic sensitivity, or the presence of virulence genes. These methods enabled linking only four isolates (112, 275, 111, and 352), which were isolated from land intensively utilized by humans—near food processing plants and in areas heavily fertilized with manure [5]. The higher identification effectiveness may be due to the fact that changes in the bacterial environment are more likely to alter the protein profile than to affect phenotypic traits.

**Figure 3:**
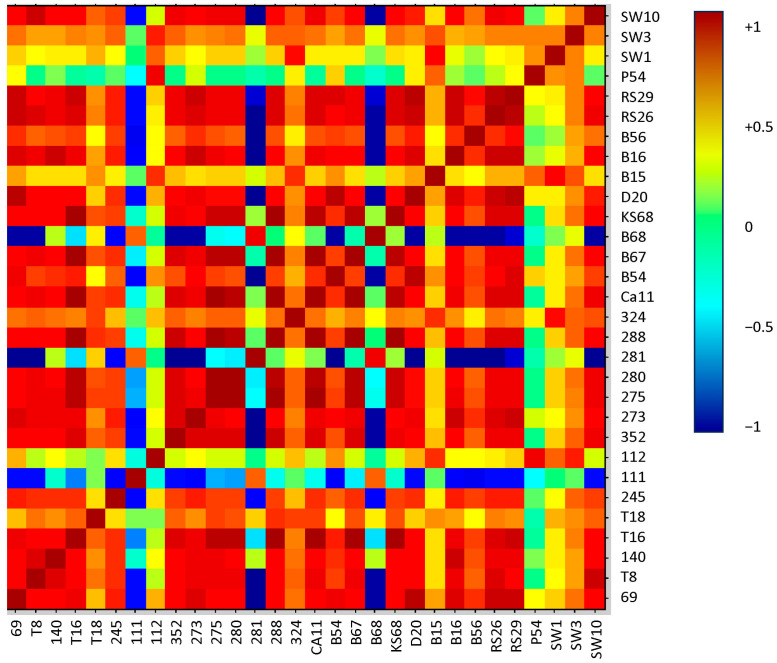
Visualization of the heat map of the data obtained from HBA (Hierarchical Binning Analysis). The heat map was generated using Metaboanalyst, indicating 30 statistically significant peaks (calculated using a t-ANOVA test) to enable distinguishing between *L. monocytogenes* isolates from four different sources: soil, plant materials (fruits and vegetables), food, and swabs from the food production environment. The dark red color indicates very close relatedness, while the blue color indicates no relatedness.

The second analysis of correlations between *L. monocytogenes* isolates from different food production environments was performed using the Composite Correlation Index (CCI) method available in the Biotyper software. The heatmap was generated from the distance matrix using the neighbor-joining method. Analyses revealed strong positive correlations (red areas) within two groups: A (288, T16, 275, 280, 288, CA11, B67, and KS68) and B (RS26, RS29, B16, 69, and 273), as well as three weaker correlations: B54 + D20, SW10 + T8, and 352 + 288 (Figure 3). Due to the same isolation environment, the analysis showed a strong correlation only between RS26 and RS29, and a weak positive correlation between 352 and B67. Unlike the dendrogram, the heatmap did not reflect the isolation environment but instead indicated similarities in phenotypic and biochemical traits. For example, group A consisted mainly of hemolysin-negative isolates, showing a positive result of the CAMP test against *S. aureus*, negative against *R. equi*, and sharing the same virulence genes. The isolates that strongly correlated in the heatmap were more often grouped based on their ability to ferment sugars and their virulence gene profiles [5]. In addition to positive correlations, the heatmap analysis revealed isolates 111, 281, and B68 that were strongly negatively correlated with most of the other isolates, as well as isolates P54 and 112, which did not correlate with most isolates, suggesting their unique phenotypic characteristics. In contrast to the other isolates, isolate 111 possessed the virulence gene *prfA*, isolate 281 exhibited resistance to the antibiotic clindamycin, and isolate B67 displayed hemolytic capability [5]. The results from previous studies confirmed that MALDI-TOF MS ribosomal proteins play key roles in determining the phenotypic characteristics of bacteria [37,38,39]. Similar analyses were conducted by Jadhav et al. [23], who demonstrated a correlation between the isolation environment and clonal lineage of dairy isolates, despite limiting the study to only two clonal lines (lineage I—16 isolates and lineage II—7 isolates). Previous studies have also validated the use of MALDI-TOF MS to differentiate *L. monocytogenes* clonal lines, although further verification with a larger group of isolates is recommended [33,40].

**Figure 4:**
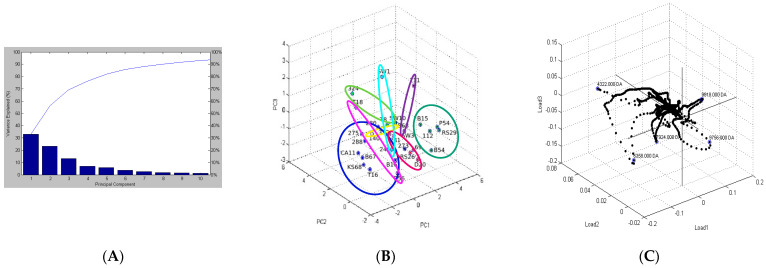
The explanation of PCA variance (**A**); PCA 3D plot (**B**) of all 30 isolates of *L. monocytogenes* (the spot represents one spectrum); and a loading plot (**C**) for the spectra of the isolates from different food production environments.

The third grouping by means of PCA explained 70% of the total variance in the spectral profiles through PC1 = 33.1%, PC2 = 23.5%, and PC3 = 13.4% (Figure 4A). Each point on the graph represents a single protein spectrum from the 30 isolates examined (Figure 4B). The points were automatically assigned to groups, labeled with the same color, and surrounded by a shape in the same color. Similar to the dendrogram, the PCA revealed that the *L. monocytogenes* isolates were located close to each other, indicating their high similarity. PCA divided the isolates into eight major groups (A–H) and two individual isolates (I and J) that were uncorrelated with the others. The greatest differences were observed between groups A and B, which were positioned on opposite sides of the graph along the PC1 axis. Group A (dark blue) included eight isolates (275, 280, 288, CA11, B16, B67, KS68, and T16), while group B (dark green) consisted of five isolates (B15, 112, P54, RS29, and B54). Between groups A and B, there was group C (red), which consisted of four isolates (281, 69, RS26, and D20). The remaining groups were as follows: D (light green: 324, T8, and SW10), E (pink: T18, B56), F (yellow: 140, B68), G (light blue: 245, SW1), and H (brown: SW3, 111).

PCA revealed that the distribution of the isolates did not directly reflect their isolation environment, which differed from the dendrogram results. The most numerous group, i.e., A, included three isolates from manure-fertilized soil, four isolates from vegetables, and one from strawberries, while the remaining groups showed no correlation with the isolation environment. Although none of the groups were distinguished by a unique phenotypic trait, the comparative analysis of phenotypic traits revealed correlations between the PCA groups and the biochemical properties of the isolates. The isolates from groups A, J, and F, located close to the PC2 axis, were more likely to exhibit β-hemolysis ability, in contrast to the isolates from groups B and H, located in the middle of the PCA plot. Additionally, groups A and B showed differences in their β-hemolytic capability, as well as in lactose, d-mannitol, and glucose degradation, which was indicative of their biochemical diversity [5]. In contrast, the isolates from groups C, E, and H were least likely to degrade d-xylose and arabinose. Thus, the PCA results may also indicate a correlation between protein profiles and phenotypic traits of the isolates studied. A comparison of the dendrogram and the PCA plot showed that PCA proved better in displaying the percentage similarity of the isolates but was less effective in mapping hierarchical relationships [41]. These results suggest that the protein profiles may be related to phenotypic traits, but their exact relationships require further research.

Further on in this study, a peptide correlation analysis, available in Biotyper, was performed to determine which peptides in the protein profiles had the greatest impact on grouping in the PCA. In Figure 4C, each individual point represents a single peak/peptide from the list of analyzed isolate spectra and indicates the extent to which the principal components relate to the original peaks. The greater the distance of the peak from the origin of the coordinate system, the greater its contribution to the variance of the dataset. The key peptides that revealed the highest correlations between their protein profiles had masses of 4296 (r = 30%, i = 6.4%); 4323 (r = 93.3%, i = 75.2%); 4876 (r = 90%, i = 16.2%); 6358 (r = 73.3%, i = 55.3%); 6390 (r = 30%, i = 37%); 6862 (r = 56.7%, i = 8.3%); 7924 (r = 56.7%, i = 27.7%); and 9750 (r = 96.7%, i = 72.5%) Da. The values in parentheses represent the average ratio (r) and intensity (i) of the respective peptides in the spectra of the 30 isolates studied. One of the main reasons for selecting these peptides was, probably, their high proportion and intensity in the protein profiles, although not all peptides with the same parameters were included in the loading plot. Three of these peptides were proposed as biomarkers for differentiating *Listeria* sp. (see Section 3.1), and another three peptides had the highest intensity in the environmental isolates. The loading plot also showed that peptide 4322 Da had the greatest impact on the correlations of the isolates distributed along the PC3 component; that peptide 6358 was essential to the variation explained by PC2; and that peptide 9756 Da was responsible for the correlations associated with the strongest PC1 component.

### 2.3. Characterization of MALDI-TOF MS Peptide Profiles of L. monocytogenes Reference Strains and Environmental L. monocytogenes Isolates

The protein profiles of environmental *L. monocytogenes* isolates differed from those of *L. monocytogenes* reference strains in terms of intensity and peptide mass distribution (Figure 1a–d). Eight *L. monocytogenes* reference strains exhibited the highest intensity (100%) with a peptide mass of 4323.1 ± 0.8 Da, while the remaining four strains (1/2b, 1/2c, 3b, and 4e) showed the most intense peptide mass of 9750.53 ± 1.3 Da (Figure 1). Three dominant peptides were identified in the environmental isolates. The first peptide, with a mass of 4323.17 ± 1.15 Da, was present in 10 isolates from soil, as well as vegetables and fruits grown in the same soil. The second peptide, with a mass of 6360.25 ± 0.4 Da, was detected in 14 of the 30 isolates tested but was absent in the protein profiles of the *L. monocytogenes* reference strains. In addition, this peptide exhibited the highest intensity (100%) in three isolates, while its intensity ranged from 24% to 80.5% in the remaining 11 isolates. The third group of peptides, with 100% intensity, had a mass of 9750.9 ± 5.1 Da, which was identical to that of the *L. monocytogenes* reference strains. Differences in the protein profiles of the reference strains and the environmental isolates were also observed in the peptide mass distribution. The isolates contained more peptides with the lowest intensity (0–2%), and their masses ranged from 9955 to 11,200 Da, compared to the reference strains, where the lowest intensity peptides had masses mainly between 7472 and 9625 Da. Jadhav et al. [23] also reported differences in peptide profiles specific to *L. monocytogenes* depending on the type of dairy products and the environment from which the strains were isolated. In turn, Rychli et al. [42] demonstrated that selected proteins in the spectra exhibited varying intensities depending on the serotype and growth conditions, whereas Zhang et al. [43] showed that the longer the incubation time of *L. monocytogenes* isolates, the greater the number of peaks in their protein profiles. This could explain the higher number of peptides in *L. monocytogenes* isolates that required 24 h longer incubation times [5]. However, some isolates derived from vegetables had fewer peptides compared to the reference strains, which excludes the effect of longer incubation times.

### 2.4. Correlation of Peptide Profiles with Phenotypic Traits

Although the spectra of the environmental isolates were very similar in both dendrogram and PCA, differences in individual peptides were still noticeable. The isolates also exhibited differences in their phenotypic characteristics [5]. Since phenotype may be partially influenced by the presence of specific peptides in the bacterial proteome, further analyses were conducted to identify potential correlations between peptide composition and selected phenotypic traits of the isolates. Understanding these correlations could provide new insights into potential biomarkers for differentiating environmental isolates.

### 2.5. Differentiating Between Reference and Environmental Strains

Statistical analysis demonstrated that *L. monocytogenes* reference strains could be differentiated from environmental isolates based on specific peptides. Only one of all peptides, with a mass of 6360.25 Da, showed a significant positive correlation (R^2^ = 0.404; *p* < 0.008) with the environmental isolates (Table 4). This peptide was present in 14 out of the 30 isolates and in none of the *L. monocytogenes* reference strains. In three isolates, it exhibited the highest intensity (100%), while in the remaining 11 isolates, its intensity ranged from 24% to 80.5%. For this reason, the 6360.25 Da peptide may serve as a potential biomarker specific to *L. monocytogenes* isolates with different phenotypic characteristics. Additionally, seven other peptides showed negative correlations, meaning they were characteristic of *L. monocytogenes* patterns but were less frequent in the environmental isolates (Table 4).

It is particularly interesting to note that as many as four peptides rarely found in environmental isolates had similar masses of 2755–2793 Da. The peptide 2755.36 Da was present in all reference strains but only in 11 isolates, while the peptide 2793.32 Da was detected in eight reference strains and only in five isolates. A similar trend was observed for peptide 2776.58 Da (present in 10 reference strains and 12 isolates) and 2782.37 Da (present in six reference strains and only in four isolates). Peptides 4361.57 Da (present in six reference strains and one isolate), 6388.02 Da (present in 10 reference strains and 7 isolates), and 7420.81 Da (present in six reference strains and two isolates) could also be used to differentiate *L. monocytogenes* reference strains from environmental isolates. These peptides exhibited intensities of at least 10% in most of the samples, indicating their significant contribution to the protein profiles of the bacteria studied. The results may, therefore, indicate that the environmental isolates, compared to the reference strains, mostly lose peptides with masses 2750–2790 in the protein spectrum and at the same time gain a peptide of 6360 Da. The results presented here align with findings from recent studies suggesting that differences in protein expression can be used to differentiate *L. monocytogenes* strains of different origins. Luciani et al. [44] conducted a proteomic analysis, identifying characteristic peptides associated with the high virulence of strains isolated from clinical cases, as compared to environmental isolates.

### 2.6. Determination of the Ability of Isolates for Hemolysis

Of the 30 *L. monocytogenes* isolates, only five isolated from vegetables (B67, B68, Ca11, P54, and KS68) exhibited β-hemolysis [5], while the remaining 25 isolates lacked this ability (Table 3). Maury et al. [45] also demonstrated that environmental isolates of *L. monocytogenes* rarely exhibited hemolysis or were completely devoid of it. Correlation analysis revealed eight statistically significant (*p* < 0.01) positive correlations between the intensity of peptides in the protein profiles and their hemolytic capability (Table 4). Peptides 2738.09 and 2776.58 Da were strongly positively correlated with hemolysis in both reference strains and *L. monocytogenes* isolates. In contrast, the peptides 2793.32 and 4361.6 Da were correlated only with hemolysis in the reference strains. Additionally, the peptides 9036.76, 9390.72, and 9750.4 Da were identified as potential biomarkers of hemolysis. These results may, therefore, indicate that the hemolytic capability may be ascribed to several peptides, the presence of which may also determine the characteristics of this trait in bacteria.

The available literature lacks studies indicating the applicability of peptides identified by the MALDI-TOF MS method as indicators of the hemolytic ability of *L. monocytogenes*. Most publications focus on conventional phenotypic methods and genetic studies, such as the detection of the *hly* gene encoding listeriolysin O [46]. Nevertheless, the results presented here, based on a large number of non-hemolytic *L. monocytogenes* isolates from various food environments, suggest that the protein profile analysis could provide new biomarkers to differentiate hemolytic from non-hemolytic isolates, which may be important for the diagnosis and epidemiology of this bacterium. A similar approach was used by Ojima-Kato et al. [47], who demonstrated that MALDI-TOF MS could differentiate *L. monocytogenes* clonal lines, while peptides could correlate with virulence properties. Therefore, this study provides the first report on the application of this technique to identify potential markers of hemolysis, particularly among hemolysin-negative isolates from diverse food production environments.

### 2.7. Determination of the Susceptibility of L. monocytogenes to Antibiotics

To determine potential biomarkers associated with antibiotic resistance in *L. monocytogenes,* a correlation analysis was performed between peptide intensity in the spectra and the diameter of the bacteria inhibition zone, in accordance with results from my previous study [5]. The higher the intensity of a peptide (IP%), the stronger its effect on antibiotic resistance, whether positive or negative, respectively. Statistical analysis identified 14 peptides with masses ranging from 4341.25 Da to 10,229.25 Da, which were significantly correlated with the diameter of bacteria inhibition zones for 14 different antibiotics (Table 5). Of these, 4 were positively correlated and 10 were negatively correlated. The strongest correlation (63%) was demonstrated for rifampicin, while the weakest correlation (43.8%) was observed for ciprofloxacin. Additionally, Table 5 provides formulas for estimating the diameter of the bacteria inhibition zone based on the intensity of the selected peptide. Although this method is not a substitute for standard reference tests, it can serve as a quick tool for the initial evaluation of a large number of isolates against the 14 most commonly used antibiotics.

The use of MALDI-TOF MS in antibiotic resistance research is gaining momentum, but most studies to date have analyzed entire spectra rather than specific peptides, as in the cases of vancomycin-resistant *Enterococcus faecium* vanB-positive strains [48], studies on the mechanism of *S. aureus* MRSA resistance [49], or the detection of carbapenemase secretion by *Bacteroides fragilis* strains [50]. Feucherolles et al. [51] demonstrated that specific protein patterns could enable rapid identification of resistant strains of *L. monocytogenes* and other foodborne pathogens. Additionally, MALDI-TOF MS, combined with peptide mass analysis (proteomic analysis of antibiotic resistance), allowed identifying key proteins in *Listeria* associated with defense mechanisms [46]. Therefore, the results presented in Table 5 provide the first report on the use of specific peptides as potential biomarkers of antibiotic resistance in *L. monocytogenes*. Further studies on the expression and functional roles of these proteins may contribute to the rapid detection of resistant strains in the food industry and clinical diagnostics.

## 3. Materials and Methods

### 3.1. Reference L. monocytogenes Strains

This study used 12 reference strains of *L. monocytogenes* with different serotypes: 1/2a (ATCC 19111), 1/2b (CIP 7832), 1/2c (ATCC 19112), 3a (ATCC 19113), 3b (CIP 7835), 3c (CIP 7836), 4a (ATCC 19114), 4b (ATCC 13932), 4c (ATCC 19116), 4d (ATCC 19117), 4e (ATCC 19118), and 7 (NCTC 10890). All strains demonstrated motility and hemolytic activity. The reference strains were used as positive controls for MALDI-TOF MS identification. External calibration of MALDI-TOF MS was performed using *E. coli* ATCC 8739, which has a list of known reference masses to enable the confirmation of system accuracy.

### 3.2. Environmental Isolates

Between 2009 and 2019, a total of 2495 samples were collected from various food production environments, including soil (*n* = 1000), fruits (*n* = 160), vegetables (*n* = 210), ready-to-eat foods (*n* = 245), meat (*n* = 400), raw fish materials (*n* = 80), salad ingredients (*n* = 120), and swabs from fish and meat processing lines (*n* = 100). These samples were tested for the presence of *L. monocytogenes*. Out of these 2495 samples, *L. monocytogenes* was isolated from 262 (10.5%). Among these, 30 isolates (11.5% of the 262) exhibited unique phenotypic and genetic characteristics compared to reference strains.

All 30 isolates exhibited no motility, and additionally, 25 of them were hemolysin-negative (Table 3). The detailed characterization of their phenotypic and genetic traits is provided in the study by Szymczak [5]. In this study, 30 *L. monocytogenes* isolates were examined. They were obtained from soil (*n* = 12), fruits (*n* = 3), vegetables (*n* = 8), ready-to-eat food (*n* = 4), and swabs from meat and fish industries (*n* = 3) (Table 3).

### 3.3. Motility, Capability for β-Hemolysis, and Sensitivity to Antibiotics

Motility was tested using soft agar (0.4%) supplemented with 2,3,5-triphenyltetrazolium chloride (TTC), and hemolytic activity was assessed on Blood Agar Base supplemented with 5% defibrinated sheep blood according to ISO 2017 guidelines. Antibiotic susceptibility testing was performed using the Kirby–Bauer disk diffusion method on Mueller–Hinton agar supplemented with 5% defibrinated horse blood and 20 mg/L of β-NAD^+^ (MH-F medium) [52]. A total of 14 antibiotics from 10 different classes, following the WHO classification [53], were tested: ampicillin (AMP, 10 µg), cephalothin (CET, 30 µg), chloramphenicol (CHL, 30 µg), ciprofloxacin (CIP, 5 µg), clindamycin (CLI, 2 µg), erythromycin (ERY, 15 µg), gentamycin (GEN, 10 µg), kanamycin (KAN, 30 µg), mezlocillin (MEZ, 30 µg), penicillin (PEN, 5 µg), rifampicin (RIF, 5 µg), streptomycin (STR, 25 µg), tetracycline (TET, 30 µg), and vancomycin (VAN, 30 µg).

### 3.4. Sample Storage and Preparation for MALDI-TOF MS

The reference strains of *L. monocytogenes*, as well as hemolysin-positive and hemolysin-negative *L. monocytogenes* isolates, were stored in a cryobank (Mast Diagnostica GmbH, Reinfeld, Germany) at −80 °C. The reference strains of *L. monocytogenes* were revived using a BHI Broth (Brain Heart Infusion) medium (Scharlau, Barcelona, Spain), into which a cryobank bead was placed, then incubated at 37 °C for 48 h, and next plated onto LSA (Listeria Selective Agar) medium and incubated at 37 °C for an additional 48 h. In turn, a modified method by Szymczak [54] was used for the revival of the environmental isolates. Briefly, the cryobank bead was placed in the BHI Broth medium with 5% defibrinated sterile sheep blood (Proanimali, Wrocław, Poland) and incubated at 37 °C for 48 h, followed by a subculture onto LSA medium (Oxoid, Basingstoke, Hampshire, UK) and incubation at 37 °C for 72 h. Finally, the resultant culture was screened on Blood Agar Base with 5% defibrinated sheep blood and incubated at 37 °C for 48 h [5].

### 3.5. Sample Preparation for MALDI-TOF MS

A standard procedure based on ethanol–formic acid extraction was adapted [55]. After overnight bacterial incubation on a BHI Agar (Oxoid, Basingstoke, United Kingdom), 20 mg of the culture was weighed into a sterile 2 mL Eppendorf tube, to which 300 µL of sterile deionized water (A&A Biotechnology, Gdańsk, Poland) was added, and the mixture was vortexed for 2 min (vortex RS-VA 10, Phoenix Instrument, Garbsen, Germany). The mixture was then combined with 900 µL of 99.8% ethanol and centrifuged at 14,000 rpm and 4 °C for 2 min (Eppendorf 5418R, Hamburg, Germany). The supernatant was removed, and 10 µL of 70% formic acid and 10 µL of acetonitrile (Merck, Darmstadt, Germany) were added, followed by centrifugation at 14,000 rpm and 4 °C for 2 min. The supernatant was transferred to a new Eppendorf tube and frozen at −32 °C.

### 3.6. MALDI-TOF MS Analysis

Prior to MALDI-TOF MS identification, the samples were thawed, centrifuged (12,000 rpm; 2 min), and then 1 µL of the extract was transferred to a MALDI-MTP Polished Steel 384 target plate (BrukerDaltonics GmbH & Co. KG, Bremen, Germany), as close as possible to the position of the BTS standard, and dried at 22 °C. Subsequently, 1 µL of an α-cyano-4-hydroxycinnamic acid (HCCA) solution (Sigma Aldrich, St. Louis, MO, USA) in 50% acetonitrile, 47.5% water, and 2.5% trifluoroacetic acid was added, and the mixture was allowed to dry at 22 °C. The MALDI-MTP plate was then placed into the microFlex LT/SH MALDI-TOF MS source. Spectra were acquired and compared to reference spectra of *L. monocytogenes* on the MALDI-TOF/TOF-MS spectrometer (Ultrafle-Xtreme; Bruker Daltonics GmbH & Co. KG, Bremen, Germany), using a laser power of 30–50% and MALDI Biotyper Offline Classification measurement mode. The spectra were analyzed using the Bruker Biotyper 3.1 BDAL (RUO) version 8.0 (DB-7854 MSP) library and FlexAnalytics 3.4 software. All measurements were performed in triplicate on the same day. All spectrometry results were externally calibrated using the Bacterial Test Standard (BTS, Bruker Daltonics), SD < 300 ppm, mass range 3.6–17 kDa—*E. coli* DH5α. Spectra were acquired in a positive linear mode, covering the mass range of *m*/*z* 2–20 kDa, using FlexControl v3.4 software (Bruker Daltonics). The entire procedure, from spectrum processing to identification, was performed automatically using the integrated pattern matching algorithm of the software. The obtained protein profiles of *L. monocytogenes* strains and isolates were added to the Bruker database, creating an in-house database, which was used for re-identification of all tested samples. After re-analyzing the spectra with the in-house library, the results were compared to the Bruker database results.

According to the criteria recommended by the manufacturer, a log (score) below 1.70 does not allow for reliable identification; a log (score) between 1.70 and 1.99 allows for the identification at the genus level; a log (score) between 2.00 and 2.29 indicates highly probable identification at the genus level and probable identification at the species level; and a log (score) between 2.30 and 3.00 indicates highly probable identification at the species level. The identification result was considered reliable when at least two top matches with a log score of 1.70–3.00 from the MALDI-TOF MS Biotyper database indicated the same species.

### 3.7. Statistical Data Analysis

The MALDI Biotyper 3.1 Offline Classification software, along with the Bruker database (MBT 7854), was used for the analysis of data from microorganism identification. A dendrogram was created by matching the main spectral peaks (MSPs) of the tested isolates. Each spectrum was compared with the others, and similarity values (Biotyper score) were used to calculate the normalized distance values between the isolates, resulting in a matching matrix shown on the dendrogram. For the heatmap, the Composite Correlation Index (CCI) values for the isolates were calculated with default settings: lower mass boundary of 3000 Da; upper boundary of 12,000 Da; mass range resolution of 4; and the number of intervals for CCI = 8. A CCI value close to +1.0 indicates a positive correlation between the isolates (dark red), values near 0 indicate no correlation (green), and a value of −1 indicates a negative correlation (dark blue). Principal Component Analysis (PCA) was performed with default settings: the lower mass boundary was set to 3000 Da; the upper boundary was set to 15,000 Da; and the mass range resolution was 2 Da. Additionally, a Pearson correlation was performed using Statistica software (StatSoft, Billerica, MA, USA, 13.1), which enabled calculating the strength (R) and significance (P) of the correlation between peptide intensity and the phenotypic characteristics of reference strains and environmental *L. monocytogenes* isolates identified in a previous study [5]. Correlations were found significant at *p* < 0.001 (very strong correlation).

## 4. Conclusions

*Listeria monocytogenes* isolates lacking motility and/or hemolytic activity are increasingly common in food production environments, making their identification with conventional methods challenging. The application of MALDI-TOF MS combined with protein extraction and an in-house database enabled accurate identification of 96.6% of isolates, compared to only 21% identified using the commercial Bruker database. Statistical analyses (dendrogram, PCA, and heat map) revealed high overall similarity among the isolates, with PCA more accurately reflecting phenotypic traits. Additionally, protein biomarkers associated with hemolytic activity and antibiotic resistance were identified. MALDI-TOF MS, coupled with an extended database, can effectively aid the detection of *L. monocytogenes* isolates with atypical phenotypic characteristics in food safety monitoring systems. Future studies will consider genome-based methods to complement these findings.

## 5. Patents

The following patents are a result of the work reported in this manuscript:

Szymczak, B. Method for determining the sensitivity of Listeria monocytogenes bacteria, especially atypical strains, to antibiotics. Notification date to the patent office: 25 November 2024, notification number: P.450342 [56].

Szymczak, B. Method for identifying typical and atypical isolates of Listeria monocytogenes bacteria capable of hemolysis. Notification date to the patent office: 30 September 2024, notification number: P.449917 [57].

## Figures and Tables

**Table 1:** Comparison of Bruker database to in-house database in the identification of *L. monocytogenes* reference strains.

Isolate IDs.	Bruker Database			In-House Database		
	Score Value (Best Match)	Score Value (Second Best Batch)	Species ID According to MALDI Biotyper 3.1	Score Value (Best Match)	Score Value (Second Best Batch)	Species ID According to MALDI Biotyper 3.1
1/2a	1.708	1.694	*L. innocua*	2.732	2.354	*L. monocytogenes* ATCC 19111
1/2b	2.263	2.214	*L. monocytogenes*	2.732	2.285	*L. monocytogenes* CIP 7832
1/2c	1.880	1.865	*L. monocytogenes*	2.655	2.324	*L. monocytogenes* ATTC 10112
3a	1.737	1.735	*L. monocytogenes*	2.685	2.374	*L. monocytogenes* ATCC 19113
3b	1.860	1.821	*L. monocytogenes*	2.724	2.412	*L. monocytogenes* CIP 7835
3c	1.902	1.788	*L. monocytogenes*	2.778	2.271	*L. monocytogenes* CIP 7836
4a	1.841	1.817	*L. monocytogenes*	2.732	2.328	*L. monocytogenes* ATCC 19114
4b	1.801	1.769	*L. monocytogenes*	2.602	2.232	*L. monocytogenes* ATCC 13932
4c	1.805	1.734	*L. monocytogenes*	2.644	2.355	*L. monocytogenes* ATCC 19116
4d	1.867	1.865	*L. monocytogenes*	2.623	2.352	*L. monocytogenes* ATCC 19117
4e	1.841	1.817	*L. innocua*	2.670	2.258	*L. monocytogenes* ATCC 19118
7	1.818	1.808	*L. monocytogenes*	2.708	2.364	*L. monocytogenes* NCTC 10890
Average	1.860	1.827		2.690	2.326	

**Table 2:** Comparison of Bruker database to in-house database in the identification of *L. monocytogenes* isolates from different food production environments.

Isolate IDs.	Bruker Database			In-House Database		
	Score Value (Best Match)	Score Value (Second Best Batch)	Species ID According to MALDI Biotyper 3.1	Score Value (Best Match)	Score Value (Second Best Batch)	Species ID According to MALDI Biotyper 3.1
69	2.245	2.117	*L. innocua*	2.419	2.255	*L. monocytogenes*
140	2.172	2.110	*L. innocua*	2.307	2.289	*L. monocytogenes*
245	1.961	1.911	*L. innocua*/*L. monocytogenes*	2.377	2.356	*L. monocytogenes*
111	1.465	1.359	Not a reliable identification	1.686	1.362	Not a reliable identification
112	2.133	2.126	*L. innocua*	2.187	2.053	*L. monocytogenes*
352	1.808	1.606	*L. monocytogenes*/not a reliable identification	2.373	2.359	*L. monocytogenes*
273	1.974	1.970	*L. innocua*	2.273	2.242	*L. monocytogenes*
275	1.895	1.825	*L. innocua*	2.265	2.088	*L. monocytogenes*
280	2.003	1.889	*L. innocua*	2.374	2.326	*L. monocytogenes*
281	1.750	1.686	*L. innocua*/not a reliable identification	2.109	2.106	*L. monocytogenes*
288	1.739	1.734	*L. innocua*/*L. seeligeri*	2.133	2.122	*L. monocytogenes*
324	2.076	2.066	*L. innocua*/*L. monocytogenes*	2.366	2.310	*L. monocytogenes*
Average for soil	1.954	1.867		2.221	2.156	
T8	2.129	2.088	*L. innocua*	2.353	2.330	*L. monocytogenes*
T16	1.908	1.888	*L. innocua*	1.723	1.662	*L. monocytogenes*
T18	1.762	1.727	*L. monocytogenes*/*L. innocua*	2.293	2.268	*L. monocytogenes*
Average for fruits	1.933	1.988		2.123	2.087	
CA11	2.028	1.994	*L. innocua*	2.353	2.247	*L. monocytogenes*
B15	1.812	1.771	*L. monocytogenes*	1.897	1.788	*L. monocytogenes*
B16	2.183	2.127	*L. monocytogenes*	2.288	2.259	*L. monocytogenes*
B54	1.994	1.968	*L. monocytogenes*	2.700	2.007	*L. monocytogenes*
B56	1.745	1.741	*L. ivanovii*/*L. innocua*	2.165	2.165	*L. monocytogenes*
B67	1.741	1.718	*L. seeligeri*/*L. innocua*	2.272	2.191	*L. monocytogenes*
B68	1.937	1.929	*L. innocua*/*L. monocytogenes*	2.369	2.315	*L. monocytogenes*
P54	2.077	2.071	*L. innocua*	1.688	1.575	*L. monocytogenes*
Average for vegetables	1.940	1.915		2.217	2.068	
KS68	1.737	1.657	*L. innocua*/not a reliable identification	2.169	2.135	*L. monocytogenes*
D20	2.047	1.967	*L. innocua*	2.008	1.874	*L. monocytogenes*
RS26	2.069	2.062	*L. innocua*	2.346	2.334	*L. monocytogenes*
RS29	2.120	2.117	*L. innocua*	2.346	2.335	*L. monocytogenes*
Average for RTE Food	1.993	1.951		2.217	2.170	
SW1	1.823	1.807	*L. innocua*	2.121	2.025	*L. monocytogenes*
SW3	1.817	1.772	*L. monocytogenes*	2.351	2.345	*L. monocytogenes*
SW10	2.040	2.028	*L. monocytogenes*	2.442	2.382	*L. monocytogenes*
Average for swabs	1.893	1.869		2.305	2.251	
All isolates average	1.947	1.900		2.218	2.137	

**Table 3:** Origin of *L. monocytogenes* isolates from different food production environments.

Environment	Sampling Locations	Isolate Number	Motility	β-Hemolysis
Soil (S)	Arable soil with natural fertilization (S1)	245	-	-
		280	-	-
		281	-	-
		288	-	-
	Garden plots from (S3)	112	-	-
		324	-	-
	Intensive cattle grazing	273	-	-
		275	-	-
	Forest from (S4)	352	-	-
		69	-	-
	The area around the meat processing plant	111	-	-
		140	-	-
Fruits	Strawberry from S1	T8	-	-
	Strawberry from S3	T16	-	-
		T18	-	-
Vegetables	Beetroot from S1	B15	-	-
		B16	-	-
		B67	-	+
		B68	-	+
	Beetroot from S3	B54	-	-
		B56	-	-
	Carrot from S1	CA11	-	+
	Potato from S3	P54	-	+
RTE food	Dumplings	D20	-	-
	Kale sprouts	KS68	-	+
	Radish sprouts	RS26	-	-
		RS29	-	-
Swabs	Seal in a hall door	SW10	-	-
	Pasteurizer belt	SW1	-	-
	Pasteurizer rollers	SW3	-	-

**Table 4:** Strength (R^2^) and significance (*p*-value) of correlations between peptide intensity and phenotypic properties of isolates from food production environments.

Biomarkers of Environmental *L. monocytogenes* Isolates		Biomarkers of Hemolysis Ability	
Peptide Mass (Da)	R^2^ and *p*-Value	Peptide Mass (Da)	R^2^ and *p*-Value
2755.36	−0.6728 *p* = 0.000	2738.09	+0.4194 *p* = 0.006
2776.58	−0.5118 *p* = 0.001	2755.36	+0.4225 *p* = 0.005
2782.37	−0.4783 *p* = 0.001	2776.58	+0.5162 *p* = 0.000
2793.32	−0.6524 *p* = 0.000	2793.32	+0.4553 *p* = 0.002
4361.57	−0.5411 *p* = 0.000	4361.60	+0.4021 *p* = 0.008
6360.25	+0.4042 *p* = 0.008	9036.76	+0.4357 *p* = 0.004
6388.02	−0.5305 *p* = 0.000	9390.72	+0.3964 *p* = 0.009
7420.81	−0.5282 *p* = 0.000	9750.40	+0.5555 *p* = 0.000

**Table 5:** Correlations between peptide intensity (PI%) in the protein profile and the diameter of the zone of inhibition by the antibiotic; correlations were calculated at a probability level of *p* < 0.001, with the exception of clindamycin and ciprofloxacin, for which a significant correlation was obtained at *p* < 0.01. Method and antibiogram results are described in Szymczak [5].

Name of Antibiotic	Mass of Peptide (Da)	Correlation Strength (R^2^)	Zone Diameter Formula of Bacterial Growth Inhibition (mm)
Gentamycin	6376.31	−0.504	diameter = 25.461 − 0.2833 × PI%
Streptomycin	7926.96	−0.602	diameter = 23.017 − 0.8574 × PI%
Kanamycin	10,229.25	−0.545	diameter = 24.099 − 11.300 × PI%
Chloramphenicol	7903.42	−0.512	diameter = 25.902 − 5.4950 × PI%
Rifampicin	6374.90	−0.630	diameter = 25.801 − 0.4315 × PI%
Cephalothin	5944.73	−0.520	diameter = 24.511 − 1.6250 × PI%
Vancomycin	9036.76	+0.612	diameter = 23.854 + 0.2672 × PI%
Clindamycin	9088.84	−0.472	diameter = 15.599 − 13.0800 × PI%
Erythromycin	9999.35	+0.541	diameter = 27.606 + 2.3420 × PI%
Ampicillin	7481.42	+0.529	diameter = 26.153 + 2.1796 × PI%
Mezlocillin	9010.29	−0.517	diameter = 28.195 − 0.2832 × PI%
Penicillin	4341.25	−0.581	diameter = 25.000 − 1.4620 × PI%
Ciprofloxacin	9390.72	−0.438	diameter = 24.276 − 1.3480 × PI%
Tetracycline	6492.85	−0.527	diameter = 22.950 − 9.6020 × PI%

## Data Availability

Data Availability Statements are available at Szymczak, B. (2024). Identification of *L. monocytogenes* and atypical isolates by MALDI TOF MS. (1–) [Dataset]. Gdańsk University of Technology. https://doi.org/10.34808/p4qc-tp95.
